# A Design Process for Preventing Brittle Failure in Strengthening RC Slabs with Hybrid FRP-HPC Retrofit Systems

**DOI:** 10.3390/ma16020755

**Published:** 2023-01-12

**Authors:** Huy Q. Nguyen, Taek Hee Han, Jun Kil Park, Jung J. Kim

**Affiliations:** 1 Department of Civil Engineering, Kyungnam University, Changwon-si 51767, Republic of Korea; 2 Coastal Development and Ocean Energy Research Center, Korea Institute of Ocean Science and Technology, 385 Haeyang-ro, Yeongdo-gu, Busan 49111, Republic of Korea

**Keywords:** CFRP, high-performance concrete, brittle failure, reinforced concrete slab, retrofit

## Abstract

The retrofitting of existing RC slabs with an innovative system comprising FRP and HPC has been demonstrated to be effective in strengthening and overcoming the logistical challenges of installation. Nonetheless, the excessive improvement of flexural strength over shear strength would cause the sudden failure of rehabilitated flexural members. The literature has previously recommended failure limits to determine the additional moment strength compared with the shear strength to prevent brittle shear failure of strengthened, continuous RC slabs. This study suggests a design process for preventing shear failure and inducing the ductile-failure mode to improve the safety and applicability of retrofitted RC slabs based on the proposed failure limits. The effectiveness of the procedure in brittle-failure prevention for the end and interior spans of retrofitted RC slabs is illustrated via a case study. The outcomes showed that the retrofit system with 0.53-mm-thick-CFRP prevented brittle failure and significantly enhanced the design-factored load and ultimate failure load by up to 2.07 times and 2.13 times, respectively.

## 1. Introduction

Rehabilitating and improving reinforced concrete (RC) structures with fiber-reinforced polymers (FRP) have recently seen enormous advances in methods and techniques thanks to widespread interest in the engineering community [1,2]. It has emerged as one of the preferred solutions to minimize environmental deterioration and save costs for upgrading existing RC infrastructure compared to constructing entirely new structures [3,4,5,6]. A wide range of literature confirmed the advantage of FRP as carbon FRP (CFRP), glass FRP (GFRP), and basalt FRP (BFRP) in repairing and enhancing RC structures due to its high tensile strength, lightweight, non-corrosion resistance, and flexible dimensions [7,8,9,10,11,12]. CFRP has superior tensile strength, whereas the others have lower material costs and poorer mechanical properties [13,14]. Despite this, relying solely on these FRP materials’ high tensile strength will cause logistical obstacles and technical difficulties. For instance, getting a well-prepared underneath surface for RC slabs or bridge decks can be challenging and expensive since traffic under overpasses cannot be interrupted, or electrical wiring, suppression systems, and ventilation ducts cannot be blocked [15,16].

**Figure 1:**
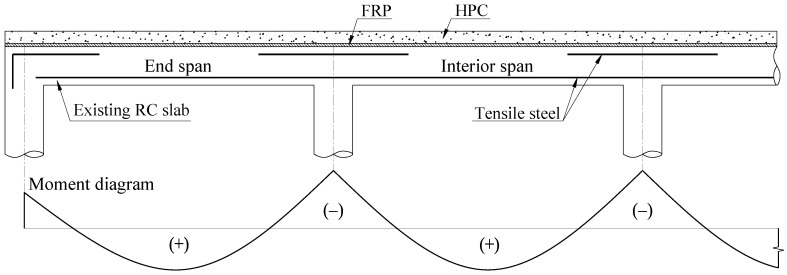
The existing RC slab with an HPC-FRP retrofit system.

In contrast, FRP installation on the top surface of slabs or bridge decks has not encountered many problems. Thus, Mosallam et al. [15] developed an innovative retrofit system made of CFRP laminate and high-performance concrete (HPC) installed on top of slabs for enhancing strength and ductility, as shown in Figure 1. The retrofitting mechanism of strengthened slabs using retrofit systems based on ACI 440.2R [17] was proposed and validated with experimental data. Nevertheless, one of the primary reasons retrofitted slabs failed to achieve their ultimate carrying capacity was a premature debonding failure between CFRP and overlay interface [18]. A specialized adhesive layer permits interfacial stress to be transferred from slab to CFRP or vice versa more effectively than from CFRP to overlay concrete, resulting in a higher potential debonding failure between overlay and CFRP [19,20]. Moon et al. [21] also suggested using shear connectors to enhance the bond strength and flexural carrying capacity of retrofitted slabs. While these attempts were valuable, the brittle shear failure and premature debonding failure of the FRP-to-concrete interface have not been sufficiently noted to yield efficient and safe structures for actual designs.

Besides, the ductility of flexural members restored by FRP is also a matter of particular concern because it can warn of ultimate failure and reduce the impact of dynamic loads [22,23,24]. In contrast with steel-reinforced concrete structures, strengthened RC structures tend to fail more brittle because of the intrinsic bond conditions between FRP and concrete [25,26,27,28,29,30]. Additionally, a sudden failure of rehabilitated flexural-continuous members would originate from an excessive enhancement in flexural strength over the shear strength. The forewarning of potential failures and preventing sudden collapses requires considering structural ductility as a critical design factor [31,32,33]. Thus, design limits to prevent shear failure and induce ductile failure of retrofitted continuous slabs were established for the end span by Nguyen et al. [34] and interior span by Kim et al. [35]. The method is used to limit the moment-carrying capacities of the strengthened slab to less than or equal to its shear-carrying capacities, which can prevent shear failure. Failure limit regions are also defined according to moment-carrying capacities to limit the added moment at the support compared to mid-span or vice versa. Even so, the difference in design limits, span lengths, and slab structures between the interior and exterior spans promotes an attempt to develop a single design guide to match the practice design purposes. The design process based on the proposed failure limits and ACI committee 440 will help to simplify a complicated and understudied problem in structural strengthening. Fortunately, it can be achieved by simply adapting the retrofit system without a violent impact on existing RC structures. CFRP and the overlay HPC significantly affect additional flexural and shear strength at the support, whereas only the HPC overlay is responsible for these properties at mid-span [36]. As a result, it is possible to adjust retrofit systems related to CFRP and overlay HPC separately or simultaneously, depending on the design purpose.

This study describes the failure limits for the end and interior span of the continuous RC slabs relevant to their bearing-carrying capacities. The limit equations for determining the design-factored load and the ultimate failure load corresponding to the failure mode are discussed. The design process for preventing brittle failure in strengthening RC slabs with hybrid FRP-HPC retrofit systems based on novel failure limit classifications is recommended. The case study is also used to illustrate the efficiency of the proposed design process. Based on the obtained result, modifications related to retrofit systems in sudden failure prevention for the end and interior spans of retrofitted RC slabs are discussed and suggested.

## 2. Overview Theory and Proposed Process

### 2.1. Failure Limits Overview

**Figure 2:**
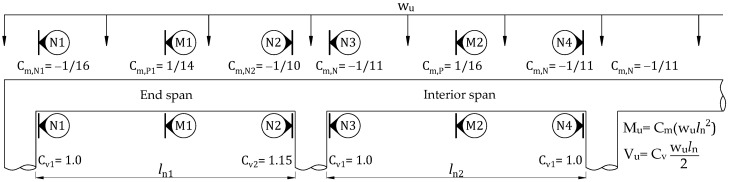
Approximate coefficients for estimating moments and shears of flexural members on continuous slabs with column supports in accordance with ACI 318M.

According to ACI 318M [37], members of continuous construction should be designed to withstand maximum-factored-load effects as defined by the elastic analysis theory. Moments and shears can be achieved by elastic analysis using the strength design method. The reasonably conservative moments and shears of flexural members subjected to the uniformly distributed load can be estimated if they have more than two spans with a longer span no greater than 1.2 times the shorter span. It can be conducted based on the applied load, length of clear span, and approximate coefficients, as shown in Figure 2.

**Figure 3:**
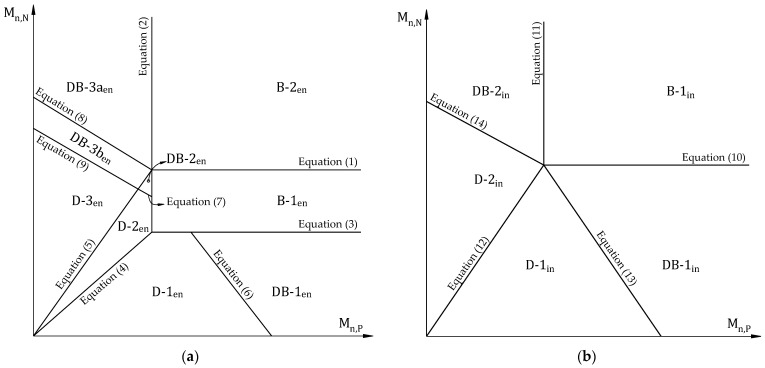
A classification of continuous RC slab failure modes based on moment and shear capacities: (**a**) the end span; (**b**) the interior span.

Nguyen et al. [34] and Kim et al. [35] established failure limits following shear limits for flexural members of frames to prevent brittle failure based on the theory of elastic analysis, as shown in Figure 3. The RC slab is considered a ductile failure when collapse occurs after forming all plastic hinges at high-internal-forces locations such as the support and mid-span. By contrast, it is supposed in brittle failure. Different failure modes according to the order of forming plastic hinges for the end and interior span of continuous RC slabs are summarized in Table 1. The proposed approach has contributed to simplifying a complex issue that prevents the sudden failure of strengthened continuous structures using external bonded FRP materials. Previous studies have demonstrated a positive effect on strengthened slabs using FRP-HPC retrofit systems [15,18,21]. We can derive the failure limits for each region of the end span from the expressions below:

(1) $$M_{n,N}=\frac{{2C}_{m,N2}}{C_{v2}}V_{n}l_{n1}$$

(2) $$M_{n,P}=\frac{{2C}_{m,P1}}{C_{v2}}V_{n}l_{n1}$$

(3) $$M_{N1}=\frac{{2C}_{m,N1}}{C_{v2}}V_{n}l_{n1}$$

(4) $$\frac{M_{N1}}{M_{n,P}}=\frac{C_{m,N1}}{C_{m,P1}}$$

(5) $$\frac{M_{n,N}}{M_{n,P}}=\frac{C_{m,N2}}{C_{m,P1}}$$

(6) $$M_{n,P}+M_{n,N}\left(\frac{C_{v2}/8+C_{m,N1}-C_{m,P1}-C_{v2}C_{m,N1}}{C_{m,N2}}+C_{v2}-1\right)=\frac{1}{4}V_{n}l_{n1}$$

(7) $$M_{n,P}\left(2C_{v2}-1\right)+M_{n,N}\left(\frac{C_{v2}/4+C_{m,P1}-C_{m,N1}-2C_{v2}C_{m,P1}}{C_{m,N2}}+1\right)=\frac{1}{2}V_{n}l_{n1}$$

(8) $$M_{n,P}\left(\frac{C_{v2}/8-C_{m,N2}}{C_{m,P1}}\right)+M_{n,N}=\frac{1}{4}V_{n}l_{n1}$$

(9) $$M_{n,P}\left(\frac{C_{v2}/4+C_{m,N2}-C_{m,N1}-2C_{v2}C_{m,N2}}{C_{m,P1}}\right)+2C_{v2}M_{n,N}=\frac{1}{2}V_{n}l_{n1}$$

Additionally, the formulas define the failure limits for each region of the interior span as follows,

(10) $$M_{n,N}=\frac{{2C}_{m,N}}{C_{v1}}V_{n}l_{n2}$$

(11) $$M_{n,P}=\frac{{2C}_{m,P}}{C_{v1}}V_{n}l_{n2}$$

(12) $$\frac{M_{n,N}}{M_{n,P}}=\frac{C_{m,N}}{C_{m,P}}$$

(13) $$M_{n,N}\left(\frac{C_{v1}/8-C_{m,P}}{C_{m,N}}\right)+M_{n,P}=\frac{1}{4}V_{n}l_{n2}$$

(14) $$M_{n,N}+M_{n,P}\left(\frac{C_{v1}/8-C_{m,N}}{C_{m,P}}\right)=\frac{1}{4}V_{n}l_{n2}$$

Based on the order to form plastic hinges, the ultimate failure loads considering plastic redistribution of the slab can be determined using the superposition method. The ultimate failure loads for the end and interior spans of continuous slabs will be calculated in accordance with Table 2.

### 2.2. Retrofitting Mechanism and Design Process Preventing Brittle Failure

**Figure 4:**
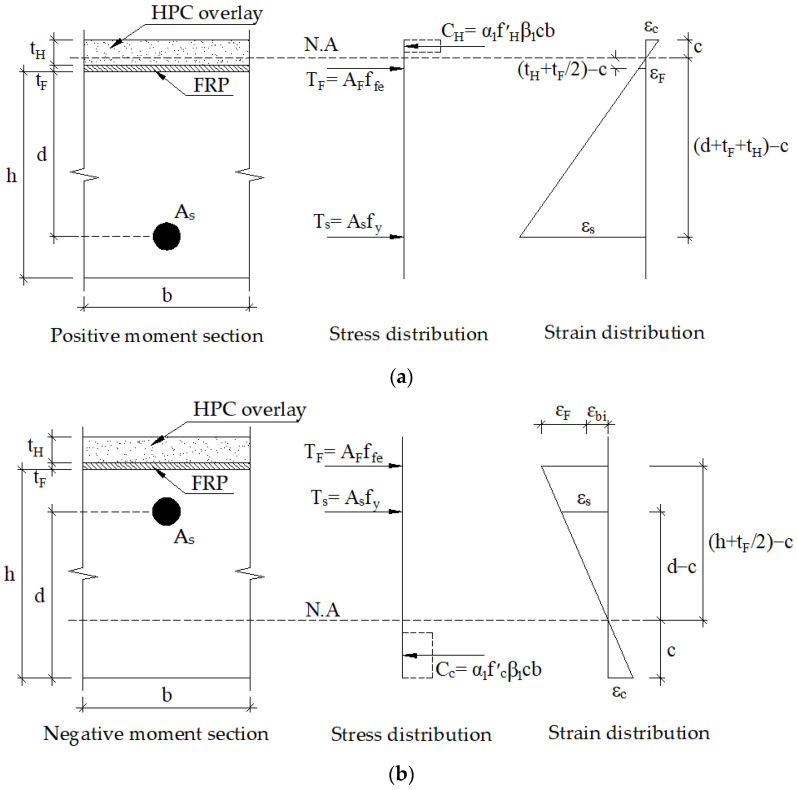
Retrofitting mechanism for a retrofitted slab (**a**) positive-moment section; (**b**) negative moment section.

The retrofitting mechanism for the strengthened slab originated from the sectional compressive force in HPC and the sectional tensile force in FRP and steel according to ACI 440.2R. For the positive moment section, it is suggested to have enough thickness to pull the neutral axis into the HPC overlay, as shown in Figure 4a. Besides, the minimum HPC thickness is recommended due to its high self-weight. For the negative moment section, the retrofitting mechanism can be predicted in a typical way, as shown in Figure 4b.

**Figure 5:**
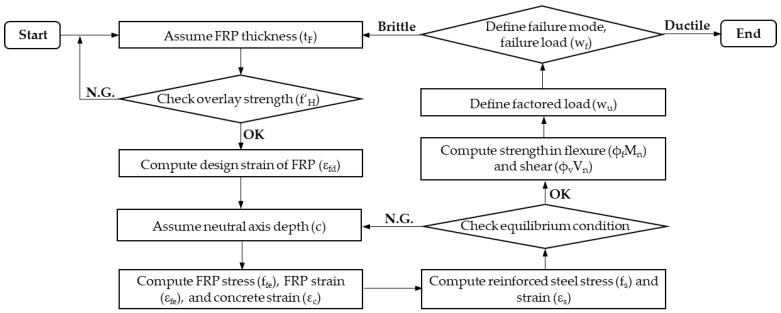
Flowchart of design for preventing brittle failure.

In this study, either evaluating the effects of long-term service loads or harsh environmental conditions is beyond the scope. The FRP sheet’s thickness, which can increase bending moments significantly of the strengthened slab, is considered a modifiable variable. The design process is based on ACI 440.2R [17] and proposed failure limits [34,35]. Before applying the design process, it is necessary to determine the input parameters related to the reference slab (i.e., *l*, h, b, A_s_, d, $f_{c}^{\prime }$, f_y_, E_s_) and retrofit system (i.e., $f_{H}^{\prime }$, $f_{\mathrm{fu}}^{*}$, E_fe_, C_E_) to perform the relevant preliminary calculations. The flowchart of design for preventing brittle failure is described in Figure 5 by the following steps:

(1) Assume FRP thickness (t_F_).
(2) The overlay strength ($f_{H}^{\prime }$) should be greater than the limits in the following equations to ensure the neutral axis within the overlay and FRP in the tensile zone [15]; otherwise, re-assume FRP thickness.
  (22) $$f_{H}^{\prime }\geq \;\frac{\varepsilon_{\mathrm{cu}}E_{\mathrm{fe}}}{1.445}{\left(\frac{t_{F}}{t_{H}}\right)}^{2}+\frac{f_{y}\left(A_{s}/b\right)}{0.7225t_{H}}$$
  (23) $$f_{H}^{\prime }\geq \;0.15f_{c}^{\prime }+\frac{\varepsilon_{\mathrm{cu}}E_{\mathrm{fe}}}{1.7}{\left(\frac{t_{F}}{t_{H}}\right)}^{2}+\frac{f_{y}\left(A_{s}/b\right)}{0.85t_{H}}$$
(3) Compute the design strain of FRP (ε_fd_) at support.
  (24) $$\varepsilon_{\mathrm{fd}}=0.41\sqrt{\frac{f_{c}^{\prime }}{{\mathrm{nE}}_{\mathrm{fe}}t_{F}}}\leq 0.9\varepsilon_{\mathrm{fu}}$$
(4) Assume the neutral axis depth (c).
(5) Compute FRP stress (f_fe_) corresponding to FRP strain (ε_fe_) and concrete strain at failure (ε_c_) by applying similar triangles based on strain compatibility.
  (25) $$f_{\mathrm{fe}}=\varepsilon_{\mathrm{fe}}E_{\mathrm{fe}}\leq f_{\mathrm{fu}}$$
  For the support section:
  (26) $$\varepsilon_{\mathrm{fe},N}=\varepsilon_{\mathrm{cu}}\left(\frac{h-c_{N}}{c_{N}}\right)-\varepsilon_{\mathrm{bi}}\leq \varepsilon_{\mathrm{fd}}$$
  (27) $$\varepsilon_{c,N}=\left(\varepsilon_{\mathrm{fe},N}+\varepsilon_{\mathrm{bi}}\right)\left(\frac{c_{N}}{h-c_{N}}\right)$$
  For the mid-span section:
  (28) $$\varepsilon_{\mathrm{fe},P}=\varepsilon_{\mathrm{cu}}\left(\frac{t_{H}-c_{P}}{c_{P}}\right)\leq \varepsilon_{\mathrm{fd}}$$
  (29) $$\varepsilon_{c,P}=\varepsilon_{\mathrm{fe},P}\left(\frac{c_{P}}{t_{H}-c_{P}}\right)$$
(6) Compute reinforced steel stress (f_s_) and strain (ε_s_).
  (30) $$f_{s}=\varepsilon_{s}E_{s}\leq f_{y}$$
  For the support section (ε_s,N_):
  (31) $$\varepsilon_{s,N}=\left(\varepsilon_{\mathrm{fe},N}+\varepsilon_{\mathrm{bi}}\right)\left(\frac{d-c_{N}}{h-c_{N}}\right)$$
  For the mid-span section (ε_s,P_):
  (32) $$\varepsilon_{s,P}=\varepsilon_{c,P}\left(\frac{{d+t}_{H}+t_{F}-c_{P}}{c_{P}}\right)$$
(7) Check the equilibrium condition by comparing c defined in Equation (33) with the value in step 4. If it is satisfied, go to step 9; otherwise, return to step 4.
  (33) $$c=\frac{A_{s}f_{s}+A_{F}f_{\mathrm{fe}}}{\alpha_{1}f_{c}^{\prime }{\;\beta }_{1}b}$$

Coefficients related to stress block (α_1_ and β_1_) are calculated as recommended in ACI 318M once concrete strain (ε_c_) is equal to or greater than the ultimate value (ε_cu_); otherwise, these values shall be estimated following the Whitney stress block, as reported by the ACI 440 committee, where $\varepsilon_{c}^{\prime }=\frac{1.7f_{c}^{\prime }}{E_{c}}$; $\beta_{1}=\frac{4\varepsilon_{c}^{\prime }-\varepsilon_{c}}{6\varepsilon_{c}^{\prime }-2\varepsilon_{c}}$; $\alpha_{1}=\frac{3\varepsilon_{c}^{\prime }\varepsilon_{c}-\varepsilon_{c}^{2}}{{3\beta }_{1}{\varepsilon^{\prime }}_{c}^{2}}$

(8) Compute strength in flexure (ϕ_f_M_n_) and shear (ϕ_v_V_n_)
  (34) $$\phi_{f}M_{n}=\phi_{f}\left(M_{\mathrm{ns}}+\psi_{f}M_{\mathrm{nf}}\right)$$
  (35) $$\phi_{v}V_{n}=\phi_{v}\left(d\sqrt{f_{c}^{\prime }}+t_{H}\sqrt{f_{H}^{\prime }}\right)\frac{b}{6}$$
  For the support section, the contribution of steel (M_ns,N_) and FRP (M_nf,N_), as
  (36) $$M_{\mathrm{ns},N}=A_{s}f_{s,N}\left(d-\frac{\beta_{1,N}c_{N}}{2}\right)$$
  (37) $$M_{\mathrm{nf},N}=A_{F}f_{\mathrm{fe},N}\left(h-\frac{\beta_{1,N}c_{N}}{2}\right)$$
  For the mid-span section, the contribution of steel (M_ns,P_) and FRP (M_nf,P_), as
  (38) $$M_{\mathrm{ns},P}=A_{s}f_{s,P}\left({d+t}_{H}+t_{F}-\frac{\beta_{1,P}c_{P}}{2}\right)$$
  (39) $$M_{\mathrm{nf},P}=A_{F}f_{\mathrm{fe},P}\left(t_{H}-\frac{\beta_{1,P}c_{P}}{2}\right)$$
(9) Define the design factored load as specified in Figure 2.
  (40) $$w_{u}=\min\left(w_{u,M};w_{u,V}\right)=\min\left(\frac{\phi_{f}M_{n}}{C_{m}l_{n}^{2}};\frac{\phi_{v}V_{n}}{C_{v}l_{n}^{}}\right)$$
(10) Define the failure mode and failure load (w_f_) in accordance with the failure limits. If the failure mode is ductile, the design process preventing brittle failure can be achieved; otherwise, re-assume FRP thickness.

## 3. Design Example

**Figure 6:**
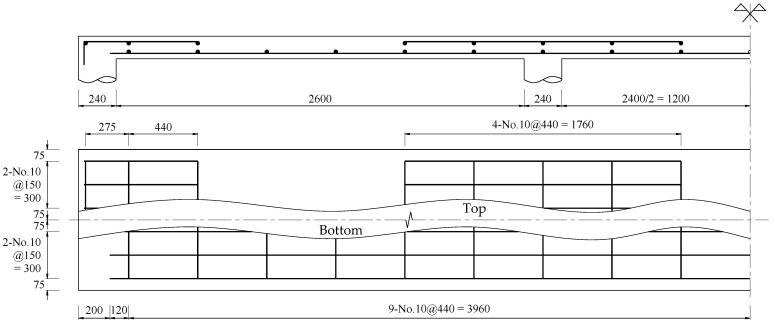
The reference slab’s reinforcement detail and dimensions (in mm).

In this case, a calculation is performed on a continuous RC slab with three spans, where the length of the end span of 2.6 m and interior span of 2.4 m are used. A uniformly distributed load is applied to the strengthened slab using the HPC-CFRP retrofit system. The moments and shears coefficients for the end and interior span are described in Figure 2. According to ACI 440 committee [17], the reduction factors of CFRP for strength contribution (ψ_f_) of 0.85 and environment of 0.95 (C_E_) are used. Besides, the flexural and shear strength reduction factors (ϕ_f_ and ϕ_v_) are 0.9 and 0.75, respectively. Material properties for the end and interior span of the continuous RC slab are given in Table 3. The reference slab’s reinforcement detail and dimensions are shown in Figure 6. The adjustable CFRP thickness with initial value and material properties of the retrofit system are provided in Table 4. For retrofit systems, shear anchors and HPC are suggested to avoid potential shear failures in overlay and premature debonding of CFRP. Previous work demonstrated that shear connectors could increase the bond strength of retrofit systems. The integrity of the retrofitted slab until reaching the ultimate bearing capacity is assumed. Table 5 shows preliminary analyses for the reference slab and retrofit system before implementing the proposed design process.

## 4. Results and Discussion

**Figure 7:**
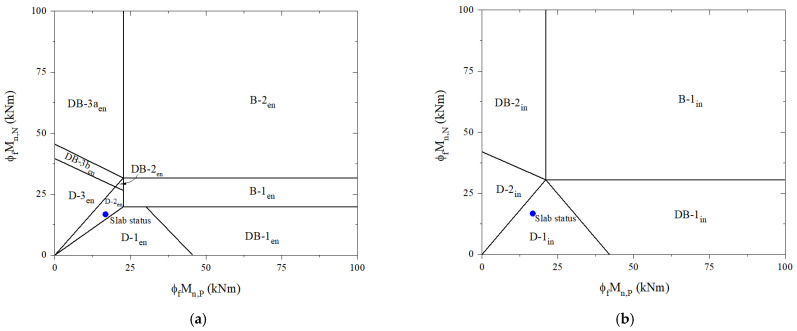
Predict failure mode based on moment-carrying capacities of the reference slab: (**a**) the end span; (**b**) the interior span.

For the reference slab, it can be seen from Table 5 that the design-factored load (w_u_) and the ultimate failure load (w_f_) for the end span are determined to be 24.7 kN/m and 31.3 kN/m, respectively. Figure 5a reveals that the failure mode is named D-2_en_. Furthermore, there is a prediction of 32 kN/m design-factored load and 39.2 kN/m ultimate failure load for the interior span with failure mode D-2_in_, as shown in Figure 7b. Before applying the design process, a preliminary analysis is performed at the adverse load-bearing section of support for the end span (section N2) and interior span (sections N3 and N4) of the reference slab and retrofit system.

**Figure 8:**
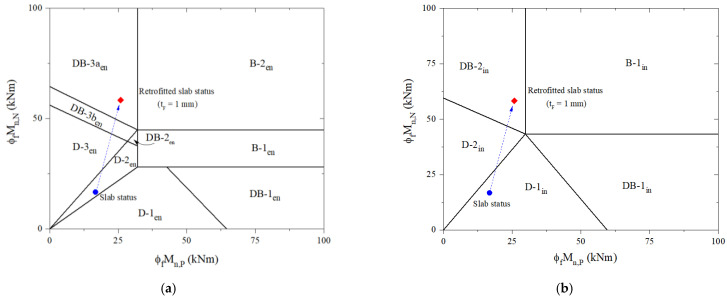
Predicting failure limits and failure mode for the retrofitted slab: (**a**) the end span; (**b**) the interior span.

For the retrofitted slab, the design process is carried out with an initial CFRP thickness of 1mm, as shown in Table 6. In step two, the compressive strength of the overlay is checked and shown that it is large enough to ensure a neutral axis in the overlay and FRP in the tensile zone. As a result of the analysis, the induction of tension in CFRP laminate at the midspan can be accomplished using a relatively low-compressive-strength concrete overlay. Nevertheless, it is preferred to use high-strength concrete to enhance the mid-span flexural strength of the slab and avoid potential shear failures of the overlay. The design strain of FRP is computed in step three before assuming the neutral axis depth in step four. The CFRP stress corresponding to its strain and concrete strain at failure is calculated in step five using similar triangles based on strain compatibility before the steel stress level is calculated in step six. According to step seven, an iterative process to achieve force equilibrium is performed as recommended by the ACI 440 committee before predicting design flexural and shear strengths in step eight. At steps nine and ten, the retrofitted slab was identified as a brittle failure for the end and interior span classified as DB-3a_en_ and DB-2_in_, respectively, as shown in Figure 8. There is an ultimate failure load at the end span of 66.38 kN/m and the interior span of 82.69 kN/m. It is not the desired outcome for the retrofitted slab to fail brittlely, regardless of the fact that its ultimate failure load is over 2.1 times that of the reference slab.

**Figure 9:**
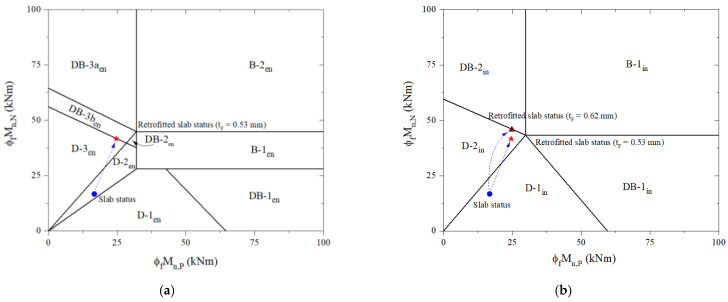
Predicting failure limits and failure mode considering brittle-failure prevention for the retrofitted slab: (**a**) the end span; (**b**) the interior span.

The failure mode of the strengthened slab is evaluated using the proposed process regarding brittle-failure prevention by adjusting CFRP thickness. For the end span of the strengthened slab, Table 6 shows the design process used to induce ductile failure. With a CFRP thickness of 0.53 mm, the retrofitted slab can fail in ductile failure mode D-3_en_, as shown in Figure 9a. There is a design-factored load of 51.05 kN/m and an ultimate failure load of 62.59 kN/m, which are higher by 2.07 times and 2.01 times, respectively, than the existing RC slab.

The interior span of the retrofitted slab is also similarly analyzed. The design procedure for preventing brittle failure can be achieved with 0.62-mm-thick CFRP laminate. Based on Table 6, the design-factored load is 68.92 kN/m. Figure 9b shows that the ductile failure mode D-2_in_ is determined to correspond to the ultimate failure load of 82.38 kN/m. Once the thickness of the CFRP laminate applied for the interior span is thicker than the end span or vice versa, it should be adjusted to be consistent with practical design requirements. With 0.53-mm-thick CFRP, the design-factored load of the strengthened slab is estimated at 68.17 k/m, which is 2.13 times higher than that of the reference slab. In accordance with Figure 9b, the failure mode remains D-2_in_ relevant to the ultimate failure load for the retrofitted slab of 76.48 kN/m, increasing by 1.97 times over the reference slab.

The proposed design process allows for the optimization of bearing capacity and increased safety required in strengthening slabs using CFRP-HPC retrofit systems. Compared with the 1-mm-thick CFRP strengthening solution, it can reduce the amount of CFRP by up to 47% without considerable changes in w_u_ and w_f_ by up to 5% and 8%, respectively, as shown in Table 6. The failure mode of the end span or the interior span will determine the failure mode of RC slabs depending on the length ratio of the end-to-interior span, slab structure, and load characteristics. Thus, it is necessary to have detailed analyses for each type of span in continuous RC slabs. The retrofit results for the end span and interior span of the strengthened RC slab are shown in Table 7.

## 5. Conclusions

The present study describes the design process for preventing brittle failure in strengthening RC slabs with hybrid FRP-HPC retrofit systems based on novel failure limit classifications. The failure limits of the end and interior span of continuous RC slabs in relation to moment- and shear- carrying capacities are discussed. The formulas for determining the design-factored load and the ultimate failure load corresponding to the failure mode are also recommended. Based on ACI 440.2R, the effectiveness of the proposed procedure of strengthened slabs using FRP-HPC retrofit systems is confirmed via the design example. The obtained results can be used to draw the following conclusions.

FRP thickness can significantly affect bearing carrying capacities and failure modes of retrofitted slabs. The case study indicates that the retrofitted slab using 0.53-mm-thick CFRP can increase the ultimate failure load by 2.13 times and fail in ductile failure mode.

The approach can optimize material strength while preventing brittle failure and reducing CFRP quantification by up to 47% without noticeable changes in bearing capacities, resulting in economic and safety benefits.

The proposed design process would encourage this strengthening technique to be applied early in practice due to its simplicity and efficiency.

The study is theoretical. Consequently, further experimental studies are recommended to confirm the suitability of the proposed method, along with evaluating the impacts of overlay regarding thickness and compressive strength, and bond strength of concrete-to-FRP interfaces.

## Figures and Tables

**Table 1:** Failure modes of continuous RC slabs according to the order of plastic hinge formation.

Span Type	Failure Modes	First Plastic Hinge	Second Plastic Hinge	Third Plastic Hinge	Shear Failure	Failure Type
End span	D-1_en_	N2	N1	M1	-	Ductile failure
	D-2_en_	N2	M1	N1	-	Ductile failure
	D-3_en_	M1	N2	N1	-	Ductile failure
	DB-1_en_	N2	N1	-	N2	Brittle failure
	DB-2_en_	N2	M1	-	N2	Brittle failure
	DB-3a_en_	M1	-	-	N2	Brittle failure
	DB-3b_en_	M1	N2	-	N2	Brittle failure
	B-1_en_	N2	-	-	N2	Brittle failure
	B-2_en_	-	-	-	N2	Brittle failure
Interior span	D-1_in_	N3, N4	M2		-	Ductile failure
	D-2_in_	M2	N3, N4		-	Ductile failure
	DB-1_in_	N3, N4	-		N3, N4	Brittle failure
	DB-2_in_	M2	-		N3, N4	Brittle failure
	B-1_in_	-	-		N3, N4	Brittle failure

**Table 2:** Calculating ultimate failure loads of continuous RC slabs.

Failure Modes	Failure Load	
D-1_en_	$w_{f}=\phi_{f}\frac{8}{l_{n1}^{2}}\left(M_{n,P}+M_{n,N}\frac{\left(1/8-C_{m,P1}\right)}{C_{m,N2}}\right)$	(15)
D-2_en_	$w_{f}=\phi_{f}\frac{4}{l_{n1}^{2}}\left(M_{n,P}+M_{n,N}\frac{\left(1/4+C_{m,N2}-C_{m,N1}-C_{m,P1}\right)}{C_{m,N2}}\right)$	(16)
D-3_en_	$w_{f}=\phi_{f}\frac{4}{l_{n1}^{2}}\left(M_{n,P}\frac{\left(1/4-C_{m,N1}\right)}{C_{m,P1}}+M_{n,N}\right)$	(17)
DB-1_en_, DB-2_en_, DB-3a_en_, DB-3b_en_, B-1_en_, B-2_en_	$w_{f}=\phi_{v}\frac{2V_{n}}{C_{v2}l_{n1}}$	(18)
D-1_in_	$w_{f}=\phi_{f}\frac{8}{l_{n2}^{2}}\left(M_{n,P}+M_{n,N}\frac{\left(1/8-C_{m,P}\right)}{C_{m,N}}\right)$	(19)
D-2_in_	$w_{f}=\phi_{f}\frac{8}{l_{n2}^{2}}\left(M_{n,P}\frac{\left(1/8-C_{m,N}\right)}{C_{m,P}}+M_{n,N}\right)$	(20)
DB-1_in_, DB-2_in_, B-1_in_	$w_{f}=\phi_{v}\frac{2V_{n}}{C_{v1}l_{n2}}$	(21)

**Table 3:** Dimensions and material properties of the existing RC slab.

Type	*l* (mm)	h (mm)	b (mm)	$f_{c}^{\prime }\;\;(\mathrm{MPa})$	A_s_ (mm^2^)	d (mm)	f_y_ (MPa)	E_s_ (GPa)
End span	2600	145	900	32	426	110	410	200
Interior span	2400	-	-	-	-	-	-	-

**Table 4:** The initial CFRP thickness and retrofit system’s material properties.

HPC Overlay		CFRP		
t_H_ (mm)	$f_{H}^{\prime }$ (MPa)	t_F_ (mm)	$f_{\mathrm{fu}}^{*}$ (MPa)	E_fe_ (GPa)
30	75	1	600	40

**Table 5:** Initial analyses of the retrofit system and reference slab.

Analysis	Reference Slab
Sectional capacity	$\phi_{f}M_{n,N}$ = 16.73 kNm; $\phi_{f}M_{n,P}$ = 16.73 kNm; $\phi_{v}V_{n}$ = 70 kN
Design factored load	For end span: $w_{u}$ = min(39.6; 24.7; 34.6; 53.8; 46.8) = 24.7 kN/m For interior span: $w_{u}$ = min(32; 46.5; 58.3) = 32 kN/m
Define failure mode and failure load	For end span: D-2_en_ according to Figure 7a; Equation (16), $w_{f}$ = 31.3 kN/m For interior span: D-1_in_ according to Figure 7b; Equation (19), $w_{f}$ = 39.2 kN/m
Self-weight	$w_{c}=\gamma_{c}\mathrm{bh}\;$ = 24(0.9)(0.145) = 3.13 kN/m
Elastic modulus	$E_{c}=4700\sqrt{f_{c}^{\prime }}$ = $4700\sqrt{32}$ = 26,600 MPa
At support, kd	kd = 24.65 mm
The crack moment at support	$I_{\mathrm{cr},N}$ = $2.78\times 10^{7}{\;\mathrm{mm}}^{4}$
The ultimate strength and strain of CFRP	$f_{\mathrm{fu}}=C_{E}f_{\mathrm{fu}}^{*}$ = 570 MPa; $\varepsilon_{\mathrm{fu}}=\frac{f_{\mathrm{fu}}^{}}{E_{\mathrm{fe}}}$ = 0.0143
Moment due to dead load	At N2 section: $M_{D,N2}$ = $\frac{1}{10}\left(3.13\right)\left(2.6^{2}\right)$ = 2.12 kNm At N3 and N4 sections: $M_{D,N3}$ = $M_{D,N4}$ = $\frac{1}{11}\left(3.13\right)\left(2.4^{2}\right)$ = 1.64 kNm
The existing state of strain	At N2 section: $\varepsilon_{\mathrm{bi}}$ = $\frac{2.12\times 10^{6}\left(145-24.65\right)}{2.78\times 10^{7}\left(26600\right)}$ = 0.00034 At N3 and N4 sections: $\varepsilon_{\mathrm{bi}}$ = $\frac{1.64\times 10^{6}\left(145-24.65\right)}{2.78\times 10^{7}\left(26600\right)}$ = 0.00027

**Table 6:** Analysis of the retrofitted slab considering brittle-failure prevention.

Process	End Span	Interior Span
1. Assume CFRP thicknesses	t_F_ = 1 mm	t_F_ = 1 mm
2. Check overlay strength	For both spans $f_{H}^{\prime }\geq \;\frac{0.003\left(40000\right)}{1.445}{\left(\frac{1}{30}\right)}^{2}+\frac{410\left(426/900\right)}{0.7225\left(30\right)}$ = 9.05 MPa (OK) $f_{H}^{\prime }\geq \;0.15\left(32\right)+\frac{0.003\left(40000\right)}{1.7}{\left(\frac{1}{30}\right)}^{2}+\frac{410\left(426/900\right)}{0.85\left(30\right)}$ = 12.49 MPa (OK)	
3. Compute the design strain of FRP at support	$\varepsilon_{\mathrm{fd}}$ = $0.41\sqrt{\frac{32}{1\left(40000\right)\left(1\right)}}$ = 0.0116 $\;\leq 0.9\varepsilon_{\mathrm{fu}}$ = 0.0128	
4. Assume neutral axis depth	At the N2 section: $c_{N}=$ 27.61 mm At mid-span section: $c_{P}=$ 10.26 mm	At support sections (N3 and N4): $c_{N}=$ 27.64 mm At mid-span section: $c_{P}=$ 10.26 mm
5. Compute FRP stress (f_fe_), FRP strain (ε_fe_), and concrete strain (ε_c_)	At the N2 section $\varepsilon_{\mathrm{fe},N}$ = $0.003\left(\frac{145-27.61}{27.61}\right)-0.00034$ = 0.0124 $\;>\varepsilon_{\mathrm{fd}}$ $\to \varepsilon_{\mathrm{fe},N}=\varepsilon_{\mathrm{fd}}$ = 0.0116 $\varepsilon_{c,N}$ = $\left(0.0116+0.00034\right)\left(\frac{27.61}{145-27.61}\right)$ = 0.0028 $f_{\mathrm{fe},N}$ = 0.0116(40,000) = 463.86 MPa At mid-span section $\varepsilon_{\mathrm{fe},P}$ = $0.003\left(\frac{30-10.26}{10.26}\right)$ = 0.0058 $\leq \varepsilon_{\mathrm{fd}}$ $\varepsilon_{c,P}$ = $0.0058\left(\frac{10.26}{30-10.26}\right)$ = 0.003 $f_{\mathrm{fe},P}$ = 0.0058(40,000) = 230.97 MPa	At support sections $\varepsilon_{\mathrm{fe},N}$ = $0.003\left(\frac{145-27.64}{27.64}\right)-0.00027$ = 0.0125 $\;>\varepsilon_{\mathrm{fd}}$ $\to \varepsilon_{\mathrm{fe},N}=\varepsilon_{\mathrm{fd}}$ = 0.0116 $\varepsilon_{c,N}$ = $\left(0.0116+0.00027\right)\left(\frac{27.64}{145-27.64}\right)$ = 0.0028 $f_{\mathrm{fe},N}$ = 0.0116(40,000) = 463.86 MPa At mid-span section (same as the end span case)
6. Compute reinforced steel stress (f_s_) and strain (ε_s_)	At the N2 section $\varepsilon_{s,N}$ = $\left(0.0116+0.00034\right)\left(\frac{110-27.61}{145-27.61}\right)$ = 0.0084 $f_{s,N}$ = 0.0084(200000) = 1680 MPa $\;>f_{y}=410\;\mathrm{MPa}$ $\to f_{s,N}=f_{y}=410\;\mathrm{MPa}$ At mid-span section $\varepsilon_{s,P}$ = $0.003\left(\frac{110+30+1-10.26}{10.26}\right)$ = 0.0382 $f_{s,P}$ = 0.0382(200,000) = 7640 MPa > $f_{y}=410\;\mathrm{MPa}$ $\to f_{s,P}=f_{y}=\;410\;\mathrm{MPa}$	At the support sections $\varepsilon_{s,N}$ = $\left(0.0116+0.00027\right)\left(\frac{110-27.64}{145-27.64}\right)$ = 0.0083 $f_{s,N}$ = 0.0083(200,000) = 1660 MPa > $f_{y}$ = 410 MPa $\to f_{s,N}=f_{y}=410\;\mathrm{MPa}$ At mid-span section (same as the end span case)
7. Check the equilibrium condition	At the N2 section, due to $\varepsilon_{c,N}<\varepsilon_{\mathrm{cu}}$ $\varepsilon_{c,N}^{\prime }$ = $\frac{1.7\left(32\right)}{26600}$ = 0.002; $\beta_{1,N}$ = $\frac{4\left(0.002\right)-0.0028}{6\left(0.002\right)-2\left(0.0028\right)}$ = 0.807 $\alpha_{1,N}$ = $\frac{3\left(0.002\right)\left(0.0028\right)-{\left(0.0028\right)}^{2}}{3\left(0.807\right){\left(0.002\right)}^{2}}$ = 0.922 $c_{N}$ = $\frac{426\left(410\right)+900\left(463.86\right)}{0.922\left(32\right)\left(0.807\right)\left(900\right)}$ = 27.61 mm (OK) At mid-span section, due to $\varepsilon_{c,P}=\varepsilon_{\mathrm{cu}}$ $\beta_{1,P}=\beta_{1}$ = 0.65; $\alpha_{1,P}=\alpha_{1}$ = 0.85 $c_{P}$ = $\frac{426\left(410\right)+900\left(230.97\right)}{0.85\left(75\right)\left(0.65\right)\left(900\right)}$ = 10.26 mm (OK)	At support sections, due to $\varepsilon_{c,N}<\varepsilon_{\mathrm{cu}}$ $\varepsilon_{c,N}^{\prime }$ = $\frac{1.7\left(32\right)}{26600}$ = 0.002; $\beta_{1,N}$ = $\frac{4\left(0.002\right)-0.0028}{6\left(0.002\right)-2\left(0.0028\right)}$ = 0.806 $\alpha_{1,N}$ = $\frac{3\left(0.002\right)\left(0.0028\right)-{\left(0.0028\right)}^{2}}{3\left(0.806\right){\left(0.002\right)}^{2}}$ = 0.923 $c_{N}$ = $\frac{426\left(410\right)+900\left(463.86\right)}{0.923\left(32\right)\left(0.806\right)\left(900\right)}$ = 27.64 mm (OK) At mid-span section (same as the end span case)
8. Compute strength in flexure and shear 8.1 Compute strength in flexure at the support section	$M_{\mathrm{ns},N}$ = $\frac{426\left(410\right)}{10^{6}}\left(110-\frac{0.807\left(27.61\right)}{2}\right)$ = 17.27 kNm $M_{\mathrm{nf},N}$ = $\frac{\left(900\times 1\right)\left(463.86\right)}{10^{6}}\left(145-\frac{0.807\left(27.61\right)}{2}\right)$ = 55.89 kNm $\phi_{f}M_{n,N}$ = 0.9[17.27+0.85(55.88)] = 58.29 kNm	$M_{\mathrm{ns},N}$ = $\frac{426\left(410\right)}{10^{6}}\left(110-\frac{0.806\left(27.64\right)}{2}\right)$ = 17.27 kNm $M_{\mathrm{nf},N}$ = $\frac{\left(900\times 1\right)\left(463.86\right)}{10^{6}}\left(145-\frac{0.806\left(27.64\right)}{2}\right)$ = 55.89 kNm $\phi_{f}M_{n,N}$ = 0.9[17.27+0.85(55.88)] = 58.29 kNm
8.2 Compute strength in flexure at the mid-span section	$M_{\mathrm{ns},P}$ = $\frac{426\left(410\right)}{10^{6}}\left(110+30+1-\frac{0.65\left(10.26\right)}{2}\right)$ = 24.04 kNm $M_{\mathrm{nf},P}$ = $\frac{\left(900\times 1\right)\left(230.97\right)}{10^{6}}\left(30-\frac{0.65\left(10.26\right)}{2}\right)$ = 5.54 kNm $\phi_{f}M_{n,P}$ = 0.9[24.04+0.85(5.54)] = 25.88 kNm	same as end span case
8.3 Compute strength in shear	$\phi_{v}V_{n}$ = $\frac{0.75}{10^{3}}\left(110\sqrt{32}+30\sqrt{75}\right)\frac{900}{6}$ = 99.23 kN	same as end span case
9. Define design factored load	$w_{u}$ = min(53.6;66.38) = 53.6 kN/m $w_{u,M}$ = min(53.6;137.96;86.23) = 53.6 kN/m $w_{u,V}$ = min(76.33;66.38) = 66.38 kN/m	$w_{u}$ = min(71.46;82.69) = 71.46 kN/m $w_{u,M}$ = min(71.46;111.32) = 71.46 kN/m $w_{u,V}$ = 82.68 kN/m
10. Define failure mode and failure load	DB-3a_en_ according to Figure 6a Equation (18), $w_{f}$ = $\frac{2\left(99.23\right)}{1.15\left(2.6\right)}$ = 66.38 kN/m	DB-2_in_ according to Figure 6b Equation (21), $w_{f}$ = $\frac{2\left(99.23\right)}{1\left(2.4\right)}$ = 82.68 kN/m
Adjust iteratively CFRP thicknesses to achieve ductile failure mode	It can be achieved with t_F_ = 0.53 mm; w_u_ = 51.05 kN/m; failure mode D-3_en_; $w_{f}$ = 62.95 kN/m, as shown in Figure 9a.	It can be achieved with t_F_ = 0.62 mm; w_u_ = 68.92 kN/m; failure mode D-2_in_; $w_{f}$ = 82.38 kN/m. However, to be consistent with the end span, t_F_ = 0.53 mm; w_u_ = 68.17 kN/m; failure mode D-2_in_; $w_{f}$ = 76.48 kN/m, as shown in Figure 9b.

**Table 7:** Summarize the retrofit results of the strengthened RC slab.

Span	Failure Mode	w_u_ (kN/m)		w_f_ (kN/m)		t_F_ (mm)
Existing end span	D-2_en_	24.70	[100%]	31.30	[100%]	-
Retrofitted end span	D-3_en_	51.05	[207%]	62.95	[201%]	0.53
Existing interior span	D-1_in_	32.00	[100%]	39.20	[100%]	-
Retrofitted interior span	D-2_in_	68.92	[215%]	82.83	[211%]	0.62
Retrofitted interior span (for consistency)	D-2_in_	68.17	[213%]	76.48	[195%]	0.53

## Data Availability

Not applicable.

## Nomenclature

A_F_, A_s_	Area of CFRP and tensile steel
b	Width of an existing slab
c	The distance between the extreme compression fiber and the neutral axis
C_E_	Environmental reduction coefficient
C_m,Ni_	Moment coefficients at support section i^th^
C_m,Pi_	Moment coefficients at mid-span section i^th^
C_vi_	Shear coefficients at section i^th^
d	The distance between the extreme compression fiber and the center of the steel
E_c_, E_s_, E_fe_	Elastic modulus of concrete, steel, and CFRP
$f_{c}^{\prime }$, $f_{H}^{\prime }$	Compressive strength concrete of existing slab and overlay
f_fe_	CFRP effective stress
f_fu_	Design ultimate strength of CFRP
$f_{\mathrm{fu}}^{*}$	FRP’s ultimate tensile strength, according to the manufacturer
f_s_	Tension steel’s stress
f_y_	Yield stress of tension steel
h	Height of an existing RC slab
I_cr_	Cracked moment
k	The ratio of the neutral axis depth to tensile steel depth measured from extreme compression fiber
*l*_ni_	Length of clear span i^th^
n	The number of CFRP layers
M_n_, V_n_	Moment and shear carrying capacity
M_n,P_, M_n,N_	Mid-span and support sections’ moment-carrying capacities
M_ns_, M_nf_	Moments contributed by tensile steel and CFRP
M_N1_	Moment-carrying capacities of the N1 section
M_D,N2_	Moment-carrying of the N-2 section
M_u_, V_u_	Factored moment and shear at sections
t_F_, t_H_	The thickness of CFRP and HPC overlay
w_f_	Ultimate failure load
w_u_	Design factored load
w_uM_, w_uV_	Design factored load follow moment and shear carrying capacities
ϕ_f_, ϕ_v_	Flexural and shear strength reduction factors
ψ_f_	CFRP strength reduction factor
α_1_, β_1_	Stress block factors
ε_bi_	Existing state strain of CFRP installation
ε_cu_, ε_fu_	Ultimate strains of concrete and CFRP
ε_fd_	Debonding strain of CFRP
ε_fe_, ε_s_	Strains of CFRP and tensile steel
γ_c_	Concrete unit weight
